# Physical Activity, Sleep, and Cognition in Preschool-Aged Children: A Scoping Review

**DOI:** 10.3390/brainsci16050436

**Published:** 2026-04-22

**Authors:** Adriana I. Ramos Marichal, Shaina P. Brady, Hsiao-Han Ho, Amanda R. Tarullo, Simone V. Gill

**Affiliations:** 1 Sargent College of Health and Rehabilitation Sciences, Boston University, 111 Cummington Mall, Boston, MA 02215, USA; airamos@bu.edu (A.I.R.M.); hsiaohan@bu.edu (H.-H.H.); 2 College of Arts and Sciences, Boston University, 64 Cummington Mall, Boston, MA 02215, USA; spbrady@bu.edu (S.P.B.); atarullo@bu.edu (A.R.T.)

**Keywords:** physical activity, sleep, cognition, preschoolers, early childhood

## Abstract

Background/Objectives: Early childhood is a critical period for executive function and broader cognitive development. Physical activity and sleep are modifiable health behaviors that support neurobiological processes underlying learning. While each has been widely examined, research investigating their combined or interactive relationships with learning remains fragmented. This scoping review synthesizes the literature on associations among physical activity, sleep, and cognition in preschool-aged children (3–5 years) and identifies gaps in the integration of these domains. Methods: Electronic databases were searched for peer-reviewed studies published within the past 10 years. Eligible studies included typically developing children aged 3–5 years and examined overlaps between at least two domains: physical activity, sleep, and cognition. Cross-sectional and longitudinal observational studies were included; intervention and review studies, and those conducted during the COVID-19 pandemic, were excluded. Results: Thirty-eight studies met the inclusion criteria. Evidence examining physical activity and sleep was limited and inconsistent. Sleep quality indicators (e.g., sleep efficiency and bedtime regularity) were more often reported to be associated with executive function and broader cognitive outcomes than total sleep duration, which showed variable relationships. Findings linking physical activity and cognition were heterogeneous; however, moderate-intensity and cognitively engaging activities were more often reported in association with executive function than total activity or intensity alone. Conclusions: Findings suggest that sleep quality and characteristics of physical activity may be relevant for preschool cognitive outcomes. Greater integration of these domains is needed, and future research should examine physical activity, sleep, and cognition within a single integrated framework to clarify potential interactive pathways linking these behaviors within this evidence base and to inform physical activity recommendations for early childhood development.

## 1. Introduction

Early childhood represents an important period for cognitive development. During this period, the brain undergoes rapid structural growth and functional development, with regions such as the prefrontal cortex maturing through changes in connectivity and organization that support emerging cognitive abilities, including the development of executive functions [1,2]. Consistent with this pattern, executive functions show marked improvements across the preschool years, reflecting substantial cognitive maturation during this stage [3]. These gains are meaningful, as individual differences in executive functions during this stage contribute to pre-academic abilities such as emergent literacy and mathematics, widely recognized as central components of school readiness [4]. Given the brain’s high level of plasticity and the rapid cognitive changes that occur in early childhood, cognitive trajectories during this period are particularly sensitive to environmental and behavioral influences, with lasting effects on learning, behavior, and development across other domains.

Physical activity and sleep are two modifiable health behaviors that play critical roles in early development. Physical activity in early childhood has been associated with a variety of favorable outcomes related to adiposity, bone and skeletal health, motor skill acquisition, psychosocial well-being, cognitive development, and cardiometabolic health [5]. In relation to cognition, engagement in physical activity has been shown to support improvements in cognitive domains such as language learning, attention, and working memory in typically developing preschoolers [6]. Similarly, adequate sleep supports neurobiological processes critical for early brain development, including memory consolidation and neural plasticity [7], whereas insufficient sleep can negatively affect physical, cognitive, and socioemotional development, with harmful downstream effects on learning, behavior, and overall well-being [8]. Together, these behaviors constitute key components of children’s daily routines that may have the potential to shape developmental trajectories during the preschool years.

Recognizing the importance of physical activity and sleep, the World Health Organization (WHO) issued movement behavior guidelines for children under 5 years of age, drawing on a 24 h Movement Behavior framework [9]. The 24 h Movement Behavior Framework conceptualizes physical activity, sedentary behavior, and sleep as interdependent behaviors that occur within 24 h, such that time allocated to one behavior displaces time allocated to another [10]. In this framework, there is a tradeoff between physical activity and sleep, though theoretically both could have positive effects on cognitive development. For children ages 3–4 years, the WHO guidelines recommend engaging in at least 180 min of physical activity spread throughout the day, including a minimum of 60 min of moderate-to-vigorous physical activity (MVPA) [9]. The guidelines further recommend obtaining 10–13 h of good-quality sleep per day, including naps, with regular sleep and wake times, and minimizing sedentary behavior by avoiding restraint for more than 1 h at a time [9]. Although sedentary behavior is a core component of the 24 h Movement Behavior framework and has been associated with cognitive outcomes, the present review focuses specifically on physical activity and sleep, given their more consistently demonstrated associations with neurobiological mechanisms underlying cognitive development. Sedentary behaviors, including screen-based media use, may occupy a substantial portion of preschoolers’ daily schedule; however, they were not examined as a separate construct in this review to maintain a focused scope on these two behavioral domains.

Although a growing body of literature has examined relationships between both physical activity and sleep with cognition, these behaviors are often studied in isolation despite their interdependence within a 24 h day. To date, the extent to which physical activity, sleep, and cognition have been examined together in studies of preschool children remains unclear. Therefore, this scoping review has two purposes. First, it aims to determine whether studies have examined physical activity, sleep, and cognition within the same study among preschool-aged children (3–5 years). Second, it seeks to examine the existing literature on pairwise associations among these domains, including studies that assess (1) physical activity and sleep, (2) sleep and cognition, and (3) physical activity and cognition. Together, these aims will help clarify the current state of the literature and identify gaps in the integration of these domains.

## 2. Methods

### 2.1. Study Design

A scoping review was conducted and reported in accordance with the Preferred Reporting Items for Systematic Reviews and Meta-Analyses extension for scoping reviews (PRISMA-ScR) checklist [11]. This approach was selected due to heterogeneity in study designs, variability in the measurement of physical activity, sleep, and cognition, and variation in cognitive outcomes across studies. Consistent with scoping review methodology, the aim was to map the extent and characteristics of the literature and to identify knowledge gaps [12].

### 2.2. Operational Definitions

For the purposes of this review, physical activity, sleep, and cognition were examined using the following operational definitions, which are summarized in Table 1.

Physical activity: Physical activity was defined as engagement in movement behaviors characterized by both duration and intensity and operationalized as time spent in total physical activity (TPA) or across intensity categories, including light physical activity (LPA) and MVPA. Studies were included if they measured physical activity either using accelerometers or with parent-reported questionnaires.

Sleep: Sleep was operationalized as sleep behavior, primarily in terms of duration and quality. Duration included total sleep duration, nighttime sleep duration, and nap duration, with an additional consideration for sleep timing (e.g., bedtime). Indicators of sleep quality were sleep efficiency, sleep latency, and sleep continuity. Studies were included if they employed parent-reported questionnaires or sleep logs, as well as accelerometry.

Cognition: Cognition was defined as children’s executive function domains, including working memory, inhibitory control, and cognitive flexibility. Broader cognitive outcomes such as school readiness, global cognitive ability, and general intellectual ability (i.e., intelligence quotient [IQ]) were also included.

### 2.3. Search Strategy

The authors collaborated with the Boston University health sciences librarian to perform a literature search across four electronic databases: PubMed, PsycInfo, Cumulative Index of Nursing and Allied Health Literature (CINAHL), and Embase. These databases were chosen to encompass biomedical, psychological, and allied health perspectives relevant to early childhood physical activity, sleep, and cognition. Search terms were developed to capture the target population and domains of interest (i.e., physical activity, sleep, and cognition). Database-specific search strings, including applied filters and controlled vocabulary, were adapted for each platform’s syntax, with complete search strategies provided in Appendix A. Broad physical activity-related terminology (e.g., physical fitness and motor activity) was used to maximize retrieval of potentially relevant studies, recognizing variability in how this domain is defined across the literature. However, final study inclusion was restricted to those examining physical activity (e.g., Studies that solely examined relationships between physical fitness and cognition were excluded). Although physical fitness and motor skills are critical aspects of children’s development, they are not aligned with operational definitions of the duration or intensity of physical activity. All search results were imported into Covidence, a web-based platform used to support study screening and selection, where duplicate records were automatically identified and removed prior to screening.

### 2.4. Eligibility Criteria

Studies were included if they met the following criteria: (1) were written in English; (2) were peer-reviewed; (3) included typically developing children aged 3–5 years; (4) examined an overlap between at least two of the three domains of interest; and (5) were published during the past 10 years. Studies employing the 24 h Movement Behavior framework were included when cognitive outcomes were assessed. Samples that extended beyond this range were retained when the mean age or a clearly defined subsample fell within the target age range.

Studies were excluded if they: (1) included children with diagnosed developmental (e.g., autism spectrum disorder), neurological (e.g., cerebral palsy), sleep (e.g., obstructive sleep apnea), behavioral (e.g., attention deficit hyperactivity disorder), or medical (e.g., cancer) conditions; (2) included typically developing children solely as a control group; (3) were classified as intervention studies, as the aim of this study was to focus on observational evidence describing naturally occurring associations; (4) were review studies, to retain focus on primary empirical studies; (5) were considered gray literature; (6) were study protocols without reported outcomes; or (7) were conducted during the COVID-19 pandemic period, due to widespread disruptions to daily routines, physical activity, sleep patterns, and early learning environments, which may limit comparability with typical developmental contexts.

### 2.5. Study Selection

**Figure 1:**
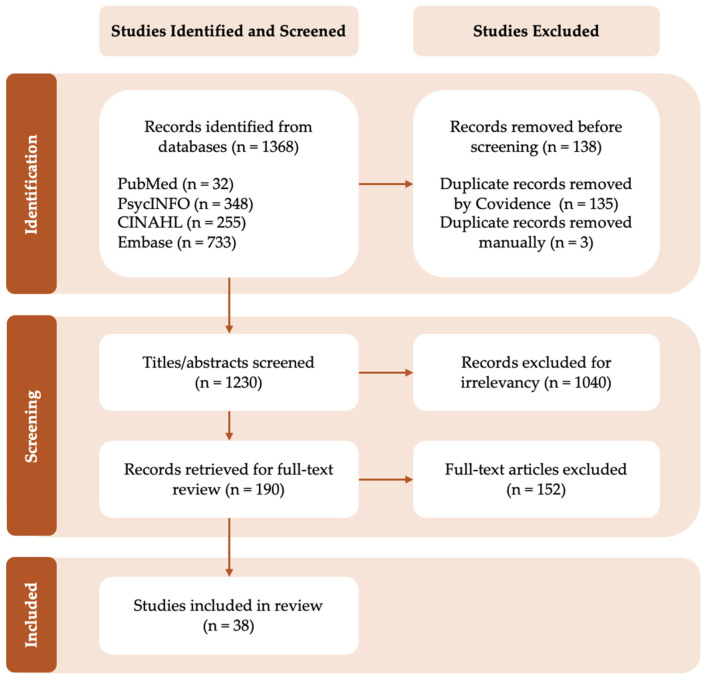
PRISMA 2020 flow diagram of study selection process.

Titles and abstracts were independently screened by two reviewers (A.I.R.M and H.H) based on the research questions and inclusion and exclusion criteria. Full-text articles were subsequently independently assessed for eligibility by two reviewers (A.I.R.M and S.V.G). Discrepancies at any stage of screening were resolved through discussion and consensus. The study selection process was documented using a PRISMA 2020 flow diagram detailing the number of records identified from each database, duplicate records removed before screening, titles and abstracts screened, records excluded for irrelevancy, records retrieved for full-text review, full-text articles excluded, and studies included in the final synthesis (Figure 1, Appendix A).

### 2.6. Data Extraction

Data from included studies were extracted and organized using a table developed by the authors. Extracted information included study design, participant age range, pairings examined, measures used, and primary findings. Data extraction was conducted independently by two reviewers, and discrepancies were resolved through discussion and consensus (A.I.R.M and S.V.G). Extracted data were used to summarize study characteristics, with key descriptive information presented in Table S1. Detailed study findings informed the synthesis presented in the Results section. Although some included studies were framed within the 24 h Movement Behavior framework, data extraction focused specifically on physical activity and sleep in relation to cognitive outcomes. Sedentary behavior and full 24-h compositional interactions were not extracted, as they were outside the scope of this review.

### 2.7. Critical Appraisal

A formal critical appraisal was not conducted. This is consistent with scoping review methodology [12], which focuses on mapping the breadth and characteristics of the existing literature rather than on evaluating methodological quality or internal validity of included studies.

## 3. Results

### 3.1. Study Characteristics

The search identified 1368 records, of which 138 duplicates were removed, leaving 1230 records for title and abstract screening. Following full-text assessment of 190 articles, 38 studies met the inclusion criteria and were included in the present scoping review (Figure 1, Appendix A). Reasons for excluding articles included study designs, patient populations, and ages that were not aligned with the inclusion criteria (e.g., an age outside of the predefined age range). Most included studies were cross-sectional (*n* = 29), with a smaller number of longitudinal investigations (*n* = 8). One study (*n* = 1) incorporated both cross-sectional and longitudinal analyses. Across included studies, physical activity, sleep, and cognition were examined in different combinations, including physical activity–sleep (*n* = 4), physical activity–cognition (*n* = 26), and sleep–cognition (*n* = 18). Note that totals exceed the number of included studies because a subset of studies adopted the 24 h Movement Behavior framework, in which physical activity and sleep were considered as a combined construct: activity. In this case, preschoolers’ activity was measured continuously to capture bouts of sleep, physical activity, and sedentary behavior. The bouts were then examined in relation to cognitive outcomes, without directly testing associations between physical activity and sleep.

### 3.2. Measurements Used

Studies included in this review used a variety of instruments to measure physical activity, sleep, and cognition. These can be found in Table S1.

Physical activity: Most studies assessed physical activity using accelerometers worn on either the hip or wrist with monitoring protocols varying across studies in terms of the number of valid wear days and daily wear-time criteria (5 days–2 weeks). In contrast, several studies incorporated parent-reported measures to capture physical activity (e.g., average amount of physical activity per day or over a period of days) and participation in organized (e.g., team sports) and non-organized (e.g., self-directed play) activities.

Sleep: Sleep was assessed using accelerometers, sleep diaries or logs, or a combination of both. Some studies solely relied on parent-reported questionnaires to obtain information on children’s sleep patterns (e.g., average daily sleep, adherence to a regular bedtime, and total naps per day).

Cognition: Studies measured cognition using a variety of standardized and task-based instruments (e.g., Early Years Toolbox, Woodcock Johnson, and Bracken School Readiness Assessment). A smaller number of studies used teacher-reported questionnaires or age-appropriate computerized tasks delivered on an iPad.

### 3.3. Findings

No studies were identified that examined physical activity, sleep, and cognition within a fully integrated analytical framework capturing relationships among all three domains. Therefore, the results focus on studies that examined pairwise associations among these domains.

#### 3.3.1. Pairwise Associations Among Physical Activity, Sleep, and Cognition

##### Physical Activity–Sleep

Sleep Duration

Few cross-sectional studies examined associations between physical activity and sleep duration, and findings were inconsistent. One study reported that longer sleep latency was associated with higher levels of physical activity (*n* = 54) [13], while another found that longer average sleep duration between participants was related to higher levels of next-day LPA (*n* = 240) [14], with both physical activity and sleep measured via accelerometry. Beyond these findings, a consistent association between physical activity and sleep duration was not identified. Engaging in more physical activity than an individual’s usual amount on a given day did not predict that night’s sleep duration [14]. Similarly, greater outdoor playtime was linked to a decreased likelihood of night waking but showed no relationship with nighttime sleep duration, with both variables measured via parent report (*n* = 497) [15].

Sleep Quality

The cross-sectional studies described above, along with one additional study, also examined associations between physical activity and sleep quality indicators; however, findings were mixed and varied depending on the analytic approach used. Greater engagement in accelerometer-measured MVPA was associated with higher nocturnal sleep efficiency and lower daytime sleepiness, both reported subjectively (*n* = 30) [16]. In contrast, between-person analyses suggested that greater average engagement in MVPA and TPA corresponded with lower sleep efficiency [14]. Conversely, higher average sleep efficiency was related to lower levels of MVPA and TPA on the following day [14]. At the within-child level, nights with higher-than-usual sleep efficiency were followed by greater MVPA and TPA [14], indicating that associations between physical activity and sleep differed depending on the unit of analysis (i.e., between versus within children).

##### Sleep–Cognition

Sleep Duration

Across included cross-sectional studies, parent-reported sleep duration was not consistently associated with cognition in preschool-aged children. Several studies (samples ranging from 217 to 2868 participants) reported no meaningful relationships between sleep duration and executive function, global cognitive ability, or language-related outcomes [17,18,19]. One study found that preschoolers who did not nap demonstrated greater working memory capacity than those who engaged in daytime napping, despite no relationship with total or nighttime sleep duration [19]. Another study similarly observed minimal associations, with weak links with inhibitory control and working memory and no relationship with cognitive flexibility (*n* = 158) [20]. Some evidence suggested a potential nonlinear relationship between sleep duration and cognition. For example, deviations from recommended sleep durations corresponded with differences in autobiographical memory specificity (i.e., ability to recall personal events), with children sleeping fewer than 10 h per night demonstrating lower specificity than those sleeping more than 10 h (*n* = 170) [21].

Sleep Quality

In contrast to sleep duration, sleep quality indicators were more consistently associated with cognitive outcomes across studies. In cross-sectional studies (samples ranging from 133 to 2868 participants), lower sleep efficiency, longer sleep onset latency, and greater sleep disruption were linked to poorer performance in executive function domains, including inhibitory control and working memory [17,22], with sleep assessed using a combination of parent-report, accelerometry, and sleep diaries. Sleep efficiency was also associated with executive function across multiple domains (i.e., inhibitory control, working memory, and cognitive flexibility) even when sleep duration showed no association with cognitive outcomes [22]. In contrast, longitudinal studies (samples ranging from 119 to 493 participants) indicated that measures of sleep timing, such as later bedtimes and greater variability in sleep schedules, were associated with lower general cognitive ability and academic skills [23,24]. Inconsistent bedtimes and disrupted sleep were further linked with poorer language skills and literacy behaviors [17].

Studies Adopting the 24 h Movement Behavior Framework

Several studies examined associations between actigraphy-assessed sleep and cognition. In a cross-sectional study using time-reallocation analysis, reallocating 15 min of MVPA to sleep corresponded with improvements in executive function (*n* = 366) [25]. In another cross-sectional study, reallocating 5–20 min of sleep to LPA or sedentary time was related to poorer executive function outcomes (*n* = 142) [26]. In a longitudinal compositional model, sleep was positively associated with cognitive flexibility (*n* = 157) [27]. In contrast, when considered alongside other movement behaviors, sleep showed less consistent associations with cognitive outcomes. For example, cross-sectional evidence indicated that sleep was negatively associated with measures of intelligence (i.e., verbal IQ, performance IQ, and full-scale IQ) compared to physical activity and sedentary time (*n* = 191) [28]. Additionally, across studies with samples ranging from 248 to 739 participants, children who met sleep recommendations demonstrated better cognitive and language-related outcomes than those who did not, in both cross-sectional and longitudinal analyses [29,30,31].

##### Physical Activity–Cognition

Executive Function

Executive function was the cognitive domain most frequently examined in relation to physical activity in preschoolers, although findings varied across domains and activity intensities. Moderate activity was more frequently reported in association with executive function than vigorous activity in a cross-sectional study (*n* = 123) [32]. For example, MVPA was positively associated with inhibitory control (*n* = 426) [33] and with cognitive flexibility in both longitudinal (parent reported) (*n* = 157) and cross-sectional (*n* = 135) studies [27,34], whereas greater engagement in vigorous activity at baseline was linked to lower levels of inhibitory control and shifting in longitudinal analyses (*n* = 317) [35], with physical activity assessed primarily via accelerometry. Similarly, accelerometer-measured LPA and TPA were generally related to executive function in cross-sectional studies (*n* = 241) [36], although longitudinal increases in MVPA were not consistently associated with changes in cognitive development (*n* = 100) [37]. However, findings were not uniform. One cross-sectional study reported that higher levels of accelerometer-measured MVPA corresponded with poorer executive function (*n* = 85) [38]. Additional cross-sectional findings indicated that working memory demonstrated a different pattern, with lower MVPA and less LPA linked to better performance (*n* = 426; *n* = 129) [33,39]. Cross-sectional evidence further suggested that associations may differ by executive function domain, with working memory corresponding to higher MVPA thresholds and inhibitory control linked to moderate activity levels (*n* = 391) [40], with physical activity assessed via accelerometry.

Broader Cognitive Outcomes

Associations between physical activity and broader cognitive and developmental outcomes were less consistent in cross-sectional studies. Objectively measured MVPA and TPA were associated with higher cognitive school readiness and self-regulation (*n* = 56) [41], although findings were mixed. For example, one study reported a weak positive relationship with self-regulation that was limited to boys (*n* = 711) [42], and further analyses indicated variations by sex and age, including links with numeracy in boys and older children and with inhibition in girls and older children [42]. Similarly, accelerometer-measured LPA was related to higher IQ scores in boys but not girls (*n* = 260) [43]. Greater versus minimal weekly participation in parent-reported physical activity was also associated with higher cognitive and linguistic development (*n* = 1870) [44].

Physical Activity Context

Beyond activity intensity, several studies examined the context in which physical activity occurred in relation to cognitive outcomes. In a cross-sectional study, outdoor play involving more dynamic movement and objectively measured via accelerometry was associated with better inhibitory control, whereas indoor play consisting primarily of more constrained or low-intensity activities, also assessed via accelerometry, showed no relationship with subsequent classroom-based executive function, potentially reflecting greater demands on behavior regulation (*n* = 72) [45]. Extending these findings to broader cognitive domains, physical activity in more engaging contexts (e.g., play with caregivers) was more consistently associated with cognitive and language outcomes than activity performed alone or in less stimulating environments [44]. Parent reports of participation in both organized and non-organized physical activity were longitudinally associated with higher intellectual ability, particularly in language development and inductive reasoning (*n* = 96) [46]. Cross-sectional findings further indicated that parent-reported non-organized physical activity and TPA were related to receptive vocabulary, with higher daily participation corresponding to higher scores (*n* = 100) [47]. The authors suggested that this may reflect caregiver-child interactions during play, as the non-organized activities assessed (e.g., going for walks, playing at the park or in the yard, and riding a bike) often involved close caregiver participation, exposing preschoolers to more words and thereby augmenting receptive vocabulary [47]. In a cross-sectional study, sports participation was also linked to better shifting (*n* = 247) [48], based on accelerometer-measured physical activity.

Studies Adopting the 24 h Movement Behavior Framework

Several studies examined associations between patterns of objectively measured physical activity and cognitive outcomes in young children. Children who adhered to physical activity recommendations demonstrated higher scores in cognitive flexibility and school readiness throughout the following year [27]. Time reallocation analyses indicated that reallocating time from LPA to MVPA corresponded with improvements in executive function in cross-sectional studies [25,26,34]. Complementing these findings, additional cross-sectional evidence indicated that children who met both physical activity and sleep recommendations demonstrated better phonological working memory and shifting performance compared to those who did not meet both recommendations, while longitudinal analyses suggested that meeting the physical activity guidelines alone at baseline was associated with better shifting performance at follow-up (12 months later) [30]. However, other cross-sectional studies with samples ranging from 374 to 858 participants reported no meaningful relationships between physical activity and cognitive outcomes [49,50]. One study further showed that reallocating time away from MVPA was associated with lower intellectual functioning compared to reallocations to MVPA [28].

## 4. Discussion

The current scoping review aimed to map the existing literature investigating associations among physical activity, sleep, and cognition in preschool-aged children, while also evaluating the extent to which these domains have been examined in combination. Although studies focusing on pairwise associations were identified, no studies examined all three domains within a single framework, capturing interrelationships among them. This highlights a key gap in the literature and suggests limited integration of these domains in research to date. When studies examine only one behavioral domain (e.g., sleep) in relation to cognition, they may potentially overestimate its contribution, given the interdependent nature of physical activity and sleep within a 24 h period. Overall, the findings across studies were heterogeneous, with inconsistent associations observed across pairings of these domains. Despite this variability, several tentative patterns were observed. There was greater convergence in findings linking sleep quality and characteristics of physical activity (e.g., intensity and context) with cognitive outcomes, whereas associations involving duration-based measures were more variable. Furthermore, few studies jointly considered physical activity and sleep, underscoring ongoing gaps in the literature.

The limited and inconsistent associations observed between physical activity and sleep align with broader evidence suggesting that links between these domains in young children are often small and variable. A recent synthesis of studies in healthy children similarly reported that greater volumes of daytime physical activity do not reliably translate into longer or more efficient sleep [51]. In the present review, associations were more often reported for sleep quality than duration, although findings were mixed. Some studies reported that greater engagement in MVPA corresponded with higher sleep efficiency, whereas others reported inverse relationships. These discrepancies may reflect differences in the timing of activity; high-intensity exercise performed closer to bedtime has been shown to induce prolonged sympathetic activation (e.g., increased heart rate) and, in turn, delay sleep onset in adults [52]. Similar mechanisms may also be relevant for children, with effects potentially differing due to developmental differences in autonomic and circadian regulation. Additionally, findings suggest that, for some children, sleep quality may influence next-day physical activity, with physical activity also influencing subsequent sleep that same night. This pattern is consistent with evidence that well-rested children, particularly those who follow bedtime routines, may demonstrate more positive mood and socioemotional behavior [53], which may, consequently, increase motivation to engage in physical activity.

Similarly, findings from this review indicate that in comparison to sleep duration, sleep quality was more often associated with cognitive functioning in preschool-aged children. Across studies, total sleep duration was often unrelated to cognitive outcomes. This inconsistency may reflect developmental changes in how much sleep children need. Sleep duration decreases with age and varies between children; the need for daytime naps gradually diminishes (e.g., from ~3.5 h at 1 month to ~1 h by 2 years) as neural networks mature and cognitive processing becomes more efficient [54,55]. As a result, across early childhood, equivalent amounts of sleep may reflect different underlying neurodevelopmental processes, potentially obscuring associations with cognition when age-related differences are not explicitly addressed. Therefore, sleep duration alone may represent an imprecise marker of the mechanisms linking sleep with cognition.

In contrast to sleep duration, findings related to sleep quality were more consistent across studies. These dimensions may reflect the restorative and organizational functions of sleep, including processes related to memory consolidation and functioning in the prefrontal cortex [56]. From a neurodevelopmental perspective, early childhood is characterized by rapid changes in sleep architecture, including evolving distributions of rapid eye movement (REM) (the stage of sleep where most dreams occur) and non-REM sleep that support synaptic plasticity, learning, and emotional regulation [7]. Longitudinal evidence further suggests that stable sleep patterns have been associated with learning, as they may relate to both concurrent levels of cognitive skills and subsequent cognitive gains during the preschool years. Children who exhibit healthier sleep habits tend to show stronger cognitive outcomes over time [57]. Together, these findings suggest that quality and consistency of sleep may be relevant indicators for cognitive functioning in early childhood, although further research is needed to clarify these relationships.

Findings from the physical activity–cognition pairing suggest that physical activity has been associated with cognitive development in early childhood, particularly executive functioning, although results were not consistent across studies. One proposed mechanism is that physical activity may be linked to the maturation of frontal brain systems via increases in cerebral blood flow, strengthening vascular networks and improving delivery of oxygen and nutrients necessary for neural functioning [58]. However, these relationships appear to vary based on developmental and contextual factors rather than intensity alone. Physiological stimulation itself may be insufficient to yield consistent cognitive benefits. Instead, the context in which physical activity occurs may influence observed associations, particularly in situations that involve rules, problem solving, or changing demands, such as team sports.

Compared to less engaging activities, research suggests that cognitively engaging exercise that combines physical effort and cognitive engagement (e.g., exergames, Simon Says, and musical chairs) has been associated with children’s executive function. However, this pattern is not consistent across all studies, likely because such tasks stimulate multiple pathways at the same time [59]. When movement is embedded within challenging or novel tasks that demand focus and quick responses, a co-activation of motor and prefrontal networks may be involved [60]. In the present review, cognitive outcomes were more often reported in association with outdoor play, caregiver involvement, and organized activities than with accumulated time, regardless of intensity. These findings suggest some consistency in the importance of activity context, although variability remains across study designs and measurement approaches. In contrast, the included studies did not identify relationships between indoor play and executive function. The type of indoor play described in these studies may have varied in the movement involved (e.g., indoor play involving cognition versus motor activities). This lack of differentiation may have attenuated potential relationships between indoor play and executive function. At the same time, reliance on activity intensity alone may contribute to the variability in findings, as time spent moving does not necessarily capture the cognitive demands of activity. Together, these findings suggest that the observed associations between physical activity and cognition may depend less on how intensely children move and more on the extent to which activities require active cognitive engagement.

Despite these insights, integration of physical activity, sleep, and cognition remains limited across studies. Research examining the 24 h Movement Behavior framework represented the most integrative approach identified in this review, as it incorporated more than one behavioral domain. These studies modeled physical activity and sleep within a 24 h day in relation to cognitive outcomes, typically using compositional time reallocation or guideline adherence approaches. In these analyses, physical activity and sleep were considered concurrently as predictors of cognition, acknowledging that time spent in one behavior displaces time spent in another. However, even within this framework, physical activity and sleep were not directly examined in relation to one another, and cognition was consistently treated as an outcome. Taken together, the current evidence base reflects partial integration of these domains, with limited examination of their potential interactive or bidirectional relationships, highlighting the need for conceptual models that consider these domains jointly rather than in isolation.

A recent narrative review in adults proposed that sleep may mediate some of the cognitive benefits of physical activity, while both behaviors may also independently influence cognition [61]. The authors further suggested that physical activity may buffer the negative effects of poor sleep, highlighting it as a potential strategy to support cognitive functioning [61]. Although this work focused on adults, its emphasis on multiple interacting pathways underscores the need for integrated studies in early childhood. Given that the preschool years represent a critical period for cognitive development, understanding potential mediating or moderating relationships among physical activity, sleep, and cognition is particularly important. Clarifying these pathways may help to inform future research examining how combinations of movement behaviors are associated with cognitive development in early childhood.

### Limitations

While this review contributes to a growing body of work examining associations among physical activity, sleep, and cognition in preschool-aged children, there are some limitations worth noting. First, the cross-sectional nature of the included studies limits conclusions about the directionality or causality of the relationships that were explored. Second, measures of physical activity, sleep, and cognition varied across studies, complicating comparisons and limiting the ability to synthesize findings across methodologies. Accelerometer- and parent-reported measures differ in accuracy, and parent-reported questionnaires may be subject to recall bias, which could influence the reported associations. Additionally, physical activity was operationalized in terms of duration or intensity, which may not fully capture other dimensions of movement behavior, such as motor competence or cognitive engagement. Finally, few studies examined potential interactions between sleep and physical activity, restricting an understanding of how these behaviors influence cognitive development. As with scoping reviews, the evidence that was synthesized is influenced by the predefined eligibility criteria and screening decisions applied, which may have resulted in the exclusion of potentially relevant studies. Also, critical appraisal of individual sources of evidence was not formally conducted, which limits the ability to evaluate the overall quality, strength, and interpretation of the evidence. This study did not focus on the role of media proliferation or sedentary behaviors. Future studies should incorporate these areas of focus in examining relationships among physical activity, sleep, and cognition in preschoolers. Addressing these limitations in future work will be important for understanding how physical activity and sleep influence cognitive outcomes in preschoolers.

## 5. Conclusions

Findings from this scoping review suggest that sleep quality and characteristics of physical activity have more often been reported in association with cognitive outcomes in preschool children. However, the evidence was heterogeneous and primarily observational. Despite a growing interest in 24 h movement behaviors, integration of physical activity, sleep, and cognition remains limited. Future research should prioritize longitudinal and mechanistic designs that examine these domains in an integrated manner, using consistent measurement approaches to clarify potential interactive and bidirectional pathways and inform holistic movement-based recommendations for early childhood cognitive development.

## Figures and Tables

**Table 1:** Operational Definitions for Sleep and Cognition.

Terms	Operational Definitions
Sleep quality indicators	
Sleep efficiency	Percentage of time spent asleep while in bed.
Sleep latency	The amount of time it takes to fall asleep once in bed.
Sleep continuity	Ability to maintain sleep throughout the night without interruptions.
Executive function domains	
Working memory	Ability to hold and manipulate information.
Inhibitory control	Ability to regulate impulses and control responses.
Cognitive flexibility	Ability to adapt behavior to changing demands.
Broader cognitive outcomes	
School readiness	Preparedness to engage successfully in formal schooling.
Global cognitive ability	Overall cognitive functioning across multiple domains (e.g., social cognition, memory ^1^, and language).

^1^ Memory under global cognition reflects general memory ability and is distinct from working memory, which is an executive function skill.

## Data Availability

The original contributions in this study are included in the article. Further inquiries can be directed to the corresponding author.

## Supplementary Materials

The following supporting information can be downloaded at: https://www.mdpi.com/article/10.3390/brainsci16050436/s1, Table S1 includes a summarization of the articles included in the review; PRISMA ScR Checklist.

## Abbreviations

The following abbreviations are used in this manuscript:

WHO	World Health Organization
MVPA	Moderate-to-vigorous physical activity
TPA	Total physical activity
LPA	Light physical activity

## Appendix A. Database Search Strategies

**Database**	**Search Strategy**
PubMed Date: 5 December 2025 Results: 32	(Child[MeSH] OR Child, Preschool[MeSH] OR preschool child*[tiab] OR preschool*[tiab] OR early childhood*[tiab] OR young child*[tiab]) AND (((Exercise[MeSH] OR Motor Activity[MeSH] OR Physical Fitness[MeSH] OR physical activit*[tiab] OR motor behavior*[tiab]) AND (Sleep[MeSH] OR sleep*[tiab])) OR ((Sleep[MeSH] OR sleep*[tiab]) AND (Cognition[MeSH] OR Executive Function[MeSH] OR cognitive function*[tiab] OR cognitive development*[tiab])) OR ((Exercise[MeSH] OR Motor Activity[MeSH] OR Physical Fitness[MeSH] OR physical activity*[tiab] OR motor behavior*[tiab]) AND (Cognition[MeSH] OR Executive Function[MeSH] OR cognitive function*[tiab] OR cognitive development*[tiab]))) Filters: English; Preschool child (2–5 years); Human; 10 years; Classical article, Dataset, Observational study, Preprint, Technical report
APAPsychInfo Date: 5 December 2025 Results: 348	(DE “Child” OR DE “Preschool Children” OR TI “preschool child*” OR TI “preschool*” OR TI “early childhood*” OR TI “young child*” OR AB “preschool child*” OR AB “preschool*” OR AB “early childhood*” OR AB “young child*”) AND (((DE “Exercise” OR DE “Motor Activity” OR DE “Physical Fitness” OR DE “Motor Behavior” OR TI “physical activit*” OR TI “exercise*” OR TI “motor activ*” OR TI “physical fitness*” OR TI “motor behavior*” OR AB “physical activit*” OR AB “exercise*” OR AB “motor activ*” OR AB “physical fitness*” OR AB “motor behavior*”) AND (DE “Sleep” OR TI “sleep*” OR AB “sleep*”)) OR ((DE “Sleep” OR TI “sleep*” OR AB “sleep*”) AND (DE “Cognition” OR DE “Executive Function” OR DE “Cognitive Development” OR TI “cognition*” OR TI “cognitive function*” OR TI “cognitive development*” OR TI “executive function*” OR AB “cognition*” OR AB “cognitive function*” OR AB “cognitive development*” OR AB “executive function*”)) OR ((DE “Exercise” OR DE “Motor Activity” OR DE “Physical Fitness” OR DE “Motor Behavior” OR TI “physical activit*” OR TI “exercise*” OR TI “motor activ*” OR TI “physical fitness*” OR TI “motor behavior*” OR AB “physical activit*” OR AB “exercise*” OR AB “motor activ*” OR AB “physical fitness*” OR AB “motor behavior*”) AND (DE “Cognition” OR DE “Executive Function” OR DE “Cognitive Development” OR TI “cognition*” OR TI “cognitive function*” OR TI “cognitive development*” OR TI “executive function*” OR AB “cognition*” OR AB “cognitive function*” OR AB “cognitive development*” OR AB “executive function*”))) Filters: English; Peer reviewed; Humans; Preschool age (2–5); Past 10 years; Empirical study, Brain imaging, Longitudinal study, Prospective study, Quantitative study
CINAHL Date: 5 December 2025 Results: 255	(MH “Child+” OR MH “Preschool Children” OR TI “preschool child*” OR TI “preschool*” OR TI “early childhood*” OR TI “young child*” OR AB “preschool child*” OR AB “preschool*” OR AB “early childhood*” OR AB “young child*”) AND (((MH “Exercise+” OR MH “Motor Activity” OR MH “Physical Fitness” OR MH “Motor Behavior” OR TI “physical activit*” OR TI “exercise*” OR TI “motor activ*” OR TI “physical fitness*” OR TI “motor behavior*” OR AB “physical activit*” OR AB “exercise*” OR AB “motor activ*” OR AB “physical fitness*” OR AB “motor behavior*”) AND (MH “Sleep+” OR TI “sleep*” OR AB “sleep*”)) OR ((MH “Sleep+” OR TI “sleep*” OR AB “sleep*”) AND (MH “Cognition” OR MH “Executive Function” OR MH “Cognitive Development” OR TI “cognition*” OR TI “cognitive function*” OR TI “cognitive development*” OR TI “executive function*” OR AB “cognition*” OR AB “cognitive function*” OR AB “cognitive development*” OR AB “executive function*”)) OR ((MH “Exercise+” OR MH “Motor Activity” OR MH “Physical Fitness” OR MH “Motor Behavior” OR TI “physical activit*” OR TI “exercise*” OR TI “motor activ*” OR TI “physical fitness*” OR TI “motor behavior*” OR AB “physical activit*” OR AB “exercise*” OR AB “motor activ*” OR AB “physical fitness*” OR AB “motor behavior*”) AND (MH “Cognition” OR MH “Executive Function” OR MH “Cognitive Development” OR TI “cognition*” OR TI “cognitive function*” OR TI “cognitive development*” OR TI “executive function*” OR AB “cognition*” OR AB “cognitive function*” OR AB “cognitive development*” OR AB “executive function*”))) Filters: English Language; Peer Reviewed; Human; Child, preschool (2–5 years); Past 10 years
Embase Date: 5 December 2025 Results: 733	(‘child’/exp OR ‘preschool child’/exp OR ‘preschool child*’:ti,ab,kw OR ‘preschool*’:ti,ab,kw OR ‘early childhood*’:ti,ab,kw OR ‘young child*’:ti,ab,kw) AND (((‘exercise’/exp OR ‘motor activity’/exp OR ‘physical fitness’/exp OR ‘motor behavior’/exp OR ‘physical activit*’:ti,ab,kw OR ‘exercise*’:ti,ab,kw OR ‘motor activ*’:ti,ab,kw OR ‘physical fitness*’:ti,ab,kw OR ‘motor behavior*’:ti,ab,kw) AND (‘sleep’/exp OR ‘sleep*’:ti,ab,kw)) OR ((‘sleep’/exp OR ‘sleep*’:ti,ab,kw) AND (‘cognition’/exp OR ‘executive function’/exp OR ‘cognitive development’/exp OR ‘cognition*’:ti,ab,kw OR ‘cognitive function*’:ti,ab,kw OR ‘cognitive development*’:ti,ab,kw OR ‘executive function*’:ti,ab,kw)) OR ((‘exercise’/exp OR ‘motor activity’/exp OR ‘physical fitness’/exp OR ‘motor behavior’/exp OR ‘physical activit*’:ti,ab,kw OR ‘exercise*’:ti,ab,kw OR ‘motor activ*’:ti,ab,kw OR ‘physical fitness*’:ti,ab,kw OR ‘motor behavior*’:ti,ab,kw) AND (‘cognition’/exp OR ‘executive function’/exp OR ‘cognitive development’/exp OR ‘cognition*’:ti,ab,kw OR ‘cognitive function*’:ti,ab,kw OR ‘cognitive development*’:ti,ab,kw OR ‘executive function*’:ti,ab,kw))) Filters: English; Human; Preschool child (1–6 years); Publication years from 2015–2026; Article, Article in press, Data papers, Preprint; Limit to terms indexed in article as ‘major focus’
TOTAL	Total Records: 1368
The asterisk (*) symbol was used to retrieve alternate word endings without typing every variation.	
