# Supplementary material for: Physical Activity, Sleep, and Cognition in Preschool-Aged Children: A Scoping Review
Source: Brain Sci. 2026 Apr 22;16(5):436. doi: 10.3390/brainsci16050436 (PMC13204363; doi:10.3390/brainsci16050436)
Supplement: Supplementary file 1 [file brainsci-16-00436-s001.zip › Table S1 (04_18_2026).pdf]

Table S1

Authors, Year	Title	Journal	Design	Sample Size, Age	Relationship Explored	Measurements Used
Abdeta et al. (2025)	Associations between 24-h movement behaviours and health in 3- and 4-year-old children from a low-income country: The SUNRISE Ethiopia study	Child: Care, Health, and Development	Cross-sectional study	n = 374<br><br>Range: 3.0–4.9 years<br><br>Mean: 4.2 (± 0.6) years	Physical activity–Cognition<br><br>Sleep–Cognition	Physical activity and sleep: Accelerometer worn on the right hip for 5 days.<br><br>Cognition: Early Years Toolbox.
Bai et al. (2021)	The relationship between physical activity, self-regulation and cognitive school readiness in preschool children	International Journal of Environmental Research and Public Health	Cross-sectional study	n = 56<br><br>Range: 3–5 years<br><br>Mean: 3.5 (± 0.69) years	Physical activity–Cognition	Physical activity: Accelerometer worn for 7 days.<br><br>Cognition: Head-Toes-Knees-Shoulders (HTKS) task and Bracken School Readiness Assessment–Third Edition (BRSA-3).
Bezerra et al. (2021)	24-hour movement behaviour and executive function in preschoolers" A	European Journal of Sport Science	Cross-sectional study	n = 123<br><br>Range: 3–5 years<br><br>Mean: 55.2 (± 9.2) months	Physical activity–Cognition<br><br>Sleep–Cognition	Physical activity: Accelerometer worn on the right hip for 7 days.<br><br>Sleep: Parent-

	compositional and isotemporal reallocation analysis					reported.<br><br><u>Cognition</u>: Early Years Toolbox.
Bezerra et al. (2023)	Biological, behavioral, and social correlates of executive function in low-income preschoolers: Insights from the perspective of the networks	Applied Neuropsychology: Child	Cross-sectional study	n = 142<br><br><u>Range:</u> 3–5 years<br><br><u>Mean:</u> 4.0 (± 0.8) years	Physical activity–Cognition	<u>Physical activity:</u> Accelerometer worn on the waist for 8 days.<br><br><u>Cognition:</u> Early Years Toolbox.
Carson et al. (2017)	Associations of subjectively and objectively measured sedentary behavior and physical activity with cognitive development in the early years	Mental Health and Physical Activity	Cross-sectional study	n = 100<br><br><u>Range:</u> 30–59 months<br><br><u>Mean:</u> 43.4 (± 9.4) months	Physical activity–Cognition	<u>Physical activity:</u> Accelerometer worn on the right hip for 7 days and parent-reported questionnaire.<br><br><u>Cognition:</u> Nebraska Barnyard task and Fish-Shark Go/No-Go task.
Chang et al. (2025)	Relationship between 24-hour movement behaviors and executive	Acta Psychologica	Cross-sectional study	n = 366<br><br><u>Range:</u> 3–6years<br><br><u>Mean:</u> 4.18 (±	Physical activity–Cognition<br><br>Sleep–Cognition	<u>Physical activity:</u> Accelerometers worn on the right hip for 7 days.

	function in preschool children based on compositional data analysis			0.87) years		Sleep: Sleep log (sleep times from the log were synchronized with the accelerometer data).<br><br>Cognition: Behavioral Rating Inventory of EF for Preschoolers (BRIEF-P).
Cliff et al. (2017)	Adherence to 24-hour movement guidelines for the early years and association with social-cognitive development among Australian preschool children	BMC Public Health	Cross-sectional study	n = 248<br><br>Range: ≥3–<6 years<br><br>Mean: 4.2 (± 0.6) years	Physical activity–Cognition<br><br>Sleep–Cognition	Physical activity: Accelerometer worn on the right hip for 7 days.<br><br>Sleep: Parent-reported.<br><br>Cognition: Four components of the Test of Emotion Comprehension battery and five-age-appropriate tasks from the global assessment used by Wellman and Liu.

Cook et al. (2019)	Associations of physical activity and gross motor skills with executive function in preschool children from low-income South African settings	Developmental Science	Cross-sectional study	n = 129<br><br><u>Range:</u> 3–6years<br><br><u>Mean:</u> 50.73 (± 8.28) months	Physical activity–Cognition	<u>Physical activity:</u> Accelerometer worn on the right hip for 7 days.<br><br><u>Cognition:</u> Early Years Toolbox.
Eythorsdottir et al. (2020)	Associations between objective measures of physical activity, sleep, and stress levels among preschool children	BMC Pediatrics	Cross-sectional study	n = 54<br><br><u>Range:</u> 2–6 years<br><br><u>Median:</u> 5.6 years (3.1–7.3)	Physical activity–Sleep	<u>Physical activity and sleep:</u> Accelerometer worn on the wrist (non-dominant wrist) for 5 days.
Garcia-Alonso et al. (2025)	Associations between physical fitness, physical activity, sedentary behavior, and executive function in preschoolers	Pediatric Research	Cross-sectional study	n = 241<br><br><u>Range:</u> 3–5 years<br><br><u>Mean:</u> 4.82 (± 0.80) years	Physical activity–Cognition	<u>Physical activity:</u> Accelerometer worn on the wrist (non-dominant wrist) for 6 days.<br><br><u>Cognition:</u> Early Years Toolbox.
Hoyniak et al. (2019)	Child sleep and socioeconomic context in the development of	Child Development	Longitudinal study	n = 493 (at recruitment)<br><br>30 months at	Sleep–Cognition	<u>Sleep:</u> Accelerometer worn on the wrist for 1–2 weeks.

	cognitive abilities in early childhood			recruitment. Assessed at 30, 36, and 42 months.		Cognition: Differential Abilities Scale.
Hoyniak et al. (2020)	Sleep across early childhood: Implications for internalizing and externalizing problems, socioemotional skills, and cognitive and academic abilities in preschool	The Journal of Child Psychology and Psychiatry	Longitudinal study	n = 119 (at recruitment)<br><br>30 months at recruitment. Assessed at 30, 36, 42, and 54 months.	Sleep–Cognition	Sleep: Accelerometer worn on the wrist for 1–2 weeks.<br><br>Cognition: Woodcock-Johnson Test of Achievement.
Kang et al. (2021)	Correlates of objectively measured sleep and physical activity among Latinx 3-to-5-year-old children	Journal of Pediatric Nursing	Cross-sectional study	n = 30<br><br>Range: 3–5-years<br><br>Mean: 45 (± 12.3) months	Physical Activity–Sleep	Physical activity and sleep: Accelerometer worn on the right hip for 5 days.<br><br>Sleep: Sleep diary and 4-item sleepiness subscale within the Pediatric Sleep Questionnaire.
Koepp et al. (2022)	Preschoolers' executive functions	Trends in Neuroscience and Education	Cross-sectional study	n = 72<br><br>Range: 3.15–6.10	Physical Activity–Cognition	Physical activity: Accelerometer worn on the

	following indoor and outdoor free play			years<br><br><u>Mean:</u> 4.42 (± 0.77) years		waist.<br><br><u>Cognition:</u> Dimensional Card Sort task, Head, Toes, Knees, and Shoulders task, and Regulation Related Skills Measure.
Lau et al. (2024)	24-Hour movement behaviors and executive functions in preschoolers: A compositional and isotemporal reallocation analysis	Child Development	Cross-sectional study	n = 426<br><br><u>Range:</u> 3–6 years<br><br><u>Mean:</u> 3.8 (± 0.6) years	Physical activity–Cognition	<u>Physical activity:</u> Accelerometer worn on the right hip for 7 days.<br><br><u>Cognition:</u> Child-friendly computerized behavioral tasks.
Lee et al. (2017)	Television viewing, reading, physical activity and brain development among young South Korean children	Journal of Science and Medicine in Sport	Cross-sectional study	n = 1870<br><br><u>Range:</u> 0–5 years	Physical activity–Cognition	<u>Physical activity:</u> Parent-reported.<br><br><u>Cognition:</u> Questionnaires developed by Kwak et al., some questions adopted from Vineland Adaptive Behavior Scales

						and Korean version of Wechsler Preschool and Primary Scale of Intelligence.
Lu et al. (2023)	Reallocation of time between preschoolers' 24-h movement behaviours and executive functions: A compositional data analysis	Journal of Sports Sciences	Cross-sectional survey	n = 135<br><br>Range: 3–5 years<br><br>Mean: 4.55 (± 0.47) years	Physical activity–Cognition<br><br>Sleep–Cognition	Physical activity: Accelerometer worn on right hip for 5 days.<br><br>Sleep: Parent-reported.<br><br>Cognition: Early Years Toolbox.
Mavilidi et al. (2025)	Adherence to 24-hour movement guidelines: Cognitive effects in Australian preschoolers	Mental Health and Physical Activity	Longitudinal study	n = 157<br><br>Range: 3–5 years<br><br>Mean: 4.48 (± 0.35) years	Physical activity–Cognition<br><br>Sleep–Cognition	Physical activity: Parent-reported.<br><br>Sleep: Parent-reported.<br><br>Cognition: Early Years Toolbox and Head-Toe-Knees-Shoulders task.
McNeill et al. (2018)	Physical activity and modified organized sport	Mental Health and Physical Activity	Observational study	n = 247<br><br>Range: 3–5 years	Physical activity–Cognition	Physical activity: Accelerometer worn on the right

	among preschool children: Associations with cognitive and psychosocial health			Mean: 4.2 (± 0.6) years		hip and 7-item parent report survey from the Longitudinal Study of Australian Children.<br><br>Cognition: Early Years Toolbox.
McNeill et al. (2020)	Compliance with the 24-Hour movement guidelines for the early years: Cross-sectional and longitudinal associations with executive function and psychosocial health in preschool children	Journal of Science and Medicine in Sport	Prospective observational study	n = 490 (at baseline), 247 (eligible for follow up), 185 (included in analytical sample)<br><br>Range: 3–5 years<br><br>Mean: 4.2 (± 0.6) years	Physical activity–Cognition<br><br>Sleep–Cognition	Physical activity: Accelerometer worn on the right hip for 7 days.<br><br>Sleep: Parent-reported.<br><br>Cognition: Early Years Toolbox.
Merín et al. (2025)	Actigraphy assessed sleep duration and quality and executive	European Child & Adolescent Psychiatry	Cross-sectional study	n = 133<br><br>Range: 3–6 years<br><br>Mean: 60.33 (± 9.04) months	Sleep–Cognition	Sleep: Accelerometer worn on the wrist (non-dominant wrist) for 7 days and 7-day sleep

	function in a sample of typically developing preschoolers					diary completed by parents.<br><br><u>Cognition:</u> Shape School task and Word Span test.
Nieto et al. (2019)	Sleep and cognitive development in preschoolers: Stress and autobiographical performance associations	Journal of Applied Developmental Psychology	Cross-sectional study	n = 170<br><br><u>Range:</u> 3–6 years<br><br><u>Mean:</u> 4.65 (± 0.93) years	Sleep–Cognition	<u>Sleep:</u> Parent-reported.<br><br><u>Cognition:</u> Autobiographical Memory Test-Preschoolers.
Nieto et al. (2022)	Relation between nighttime sleep duration and executive functioning in a nonclinical sample of preschool children	Scandinavian Journal of Psychology	Cross-sectional study	n = 158<br><br><u>Range:</u> 38–78 months<br><br><u>Mean:</u> 56.35 (± 11.24) months	Sleep–Cognition	<u>Sleep:</u> Parent-reported.<br><br><u>Cognition:</u> Shape School task, Word Span tasks (Word Span Forward and Words Span Backward), and Spanish adaptation of the vocabulary subtest from the Wechsler Preschool and Primary Scale of Intelligence.

O'Connor et al. (2020)	Association of Lifestyle Factors and Neuropsychological Development of 4-Year-Old Children	International Journal of Environmental Research and Public Health	Cross-sectional study	n = 1650<br><br>4 years	Physical activity–Cognition<br><br>Sleep–Cognition	Physical activity: Parent-reported.<br><br>Sleep: Parent-reported.<br><br>Cognition: Spanish version of the McCarthy Scales of Children's Abilities.
Quan et al. (2018)	Preschoolers' Technology-Assessed Physical Activity and Cognitive Function: A Cross-Sectional Study	Journal of Clinical Medicine	Cross-sectional study	n = 260<br><br>Mean: 57.2 (± 5.4) months	Physical activity–Cognition	Physical activity: Accelerometer worn on the right hip for 7 days.<br><br>Cognition: Short form of the Chinese version of Wechsler Young Children Scale of Intelligence.
Ramos-Munell et al. (2025)	Striking the right balance of daily 24-h physical behaviors in preschoolers	Journal of Public Health	Cross-sectional study	n = 391<br><br>Range: 2–6 years<br><br>Mean: 4.71 (± 0.86) years	Physical activity–Cognition<br><br>Sleep–Cognition	Physical activity and sleep: Accelerometer worn on the waist for 7 days.<br><br>Cognition: iPad games (Go/No-Go

						and Mr. Ant).
Schlieber & Han (2021)	The sleeping patterns of Head Start children and the influence on developmental outcomes	Child: Care, Health and Development	Cross-sectional study	n = 2,868<br><br>Range: 3–4	Sleep–Cognition	Sleep: Parent-reported.<br><br>Cognition: Peabody Picture Vocabulary Test-IV, Expressive One-Word Picture Vocabulary Test, subtests of the Woodcock-Johnson III, and teacher-reported assessments.
St. Laurent et al. (2022)	Temporal relationships between device-derived sedentary behavior, physical activity, and sleep in early childhood	Sleep Research Society	Observational study	n = 240<br><br>Range: 33–71 months<br><br>Mean: 50.8 (± 9.8) months	Physical activity–Sleep	Physical activity and sleep: Accelerometer worn on the wrist (non-dominant wrist) for 9 days.<br><br>Sleep: Sleep diary.
Vabø et al. (2022)	The multivariate physical activity signatures associated with self-regulation, executive function, and	Frontiers in Psychology	Cross-sectional study	n = 711<br><br>Range: 3–5 years<br><br>Mean: 4.6 (± 0.8) years	Physical activity–Cognition	Physical activity: Accelerometer worn on the right hip for 7 days.<br><br>Cognition: Early Years Toolbox

	early academic learning in 3-5-year-old children					(EYT).
Vanderloo et al. (2022)	Association between physical activity, screen time and sleep, and school readiness in Canadian children aged 4 to 6 years	Journal of Developmental & Behavioral Pediatrics	Prospective cohort study	n = 739<br><br>Range: 4–6 years<br><br>Mean: 5.9 (± 0.12) years	Physical activity–Cognition<br><br>Sleep–Cognition	Physical activity and sleep: Parent-reported.<br><br>Cognition: Early Development Instrument.
Vanhala et al. (2024)	Developmental associations of fundamental motor skills and executive functions in preschoolers — The role of the physical activity and the effects on early numeracy	Trends in Neuroscience and Education	Longitudinal study	n = 317<br><br>Range: 3–6 years<br><br>Mean: 4.5 years (T1), 5.4 years (T2), 6.4 (T3)	Physical activity–Cognition	Physical activity: Accelerometer worn on the right hip over 7 days.<br><br>Cognition: Computer-based tests.
Verswijveren et al. (2020)	Longitudinal associations of sedentary time and physical activity duration and patterns with cognitive	Mental Health and Physical Activity	Longitudinal prospective cohort study	n = 100 (at baseline)<br><br>Range: 2.5–5.0. years	Physical activity–Cognition	Physical activity: Accelerometer worn on the right hip for 7 days.<br><br>Cognition: Fish-Shark

	development in early childhood					Go/No-Go task, Nebraska Barnyard task, and Woodcock-Johnson III test battery.
Willoughby et al. (2018)	Testing the association between physical activity and executive function skills in early childhood	Early Childhood Research Quarterly	Cross-sectional study	n = 85<br><br><u>Range:</u> 3–5 years<br><br><u>Mean:</u> 4.4 (± 0.7) years	Physical activity–Cognition	<u>Physical activity:</u> Accelerometer worn on the right hip.<br><br><u>Cognition:</u> EF Touch (computerized battery of EF tasks).
Xu et al. (2025)	24-hour movement behaviors and cognitive ability in preschool children: A compositional and isotemporal reallocation analysis	PLOS One	Cross-sectional study	n = 191<br><br><u>Range:</u> 3-6 years<br><br><u>Mean:</u> 4.59 (± 0.99)	Physical activity–Cognition<br><br>Sleep–Cognition	<u>Physical activity:</u> Accelerometer worn on the right hip for 7 days.<br><br><u>Sleep:</u> Parent-reported and sleep log.<br><br><u>Cognition:</u> Short form of the Chinese version of the Wechsler Young Children Scale of Intelligence.

Xu et al. (2016)	Associations of outdoor play and screen time with nocturnal sleep duration and pattern among young children	Acta Paediatrica	Cross-sectional and longitudinal study	n = 497, 415 and 369 mother–child dyads retained at ages 2, 3.5 and five years, respectively<br><br><u>Range:</u> 2–5 years	Physical activity–Sleep	<u>Physical activity:</u> Parent-reported.<br><br><u>Sleep:</u> Parent-reported (based on questionnaire from the Prevention of Overweight in Infancy study.
Zahran et al. (2024)	Optimal levels of sleep, sedentary behaviour, and physical activity needed to support cognitive function in children of the early years	BCM Pediatrics	Cross-sectional study	n = 858<br><br><u>Range:</u> 2.8–5.5 years<br><br><u>Mean:</u> 4.2 (± 0.7) years	Physical activity–Cognition<br><br>Sleep–Cognition	<u>Physical activity and sleep:</u> Accelerometer worn on the right hip.<br><br><u>Cognition:</u> Early Years Toolbox.
Zhang et al. (2022)	Longitudinal associations of subjectively-measured physical activity and screen time with cognitive development in young children	Mental Health and Physical Activity	Prospective longitudinal cohort study	n = 96<br><br><u>Range:</u> 2.5–5.0<br><br><u>Mean:</u> 3.69 (± 0.78) years	Physical activity–Cognition	<u>Physical activity:</u> Parent-reported.<br><br><u>Cognition:</u> Fish-Shark Go/ No-Go task, Nebraska Barnyard task, and tests from Woodcock-Johnson III battery.

Zhang et al. (2021)	Associations between sleep duration, adiposity indicators, and cognitive development in young children	Sleep Medicine	Cross-sectional study	n = 217<br><br>Range: 19–60 months<br><br>Mean: 35.51(± 9.59) months	Sleep–Cognition	Sleep: Parent-reported.<br><br>Cognition: Early Years Toolbox
